# The Management of Brain Metastases in Non-Small Cell Lung Cancer

**DOI:** 10.3389/fonc.2014.00248

**Published:** 2014-09-15

**Authors:** Scott Owen, Luis Souhami

**Affiliations:** ^1^Division of Medical Oncology, Department of Oncology, McGill University Health Centre, Montreal, QC, Canada; ^2^Division of Radiation Oncology, Department of Oncology, McGill University Health Centre, Montreal, QC, Canada

**Keywords:** brain metastases, lung cancer, targeted therapy, radiation therapy, chemotherapy, stereotactic radiosurgery, surgery

## Abstract

Brain metastases (BM) are a common and lethal complication of non-small cell lung cancer (NSCLC), which portend a poor prognosis. In addition, their management implies several challenges including preservation of neurological and neurocognitive function during surgery or radiation-therapy, minimizing iatrogenic complications of supportive medications, and optimizing drug delivery across the blood–brain barrier. Despite these challenges, advancements in combined modality approaches can deliver hope of improved overall survival and quality of life for a subset of NSCLC patients with BM. Moreover, new drugs harnessing our greater understanding of tumor biology promise to build on this hope. In this mini-review, we revised the management of BM in NSCLC including advancements in neurosurgery, radiation therapy, as well as systemic and supportive therapy.

## Introduction

Lung cancer is the leading cause of cancer mortality worldwide, accounting for 1.38 million annual deaths, representing 18.2% of total deaths from cancer (1). Among those, approximately 7.4% of non-small cell lung cancer (NSCLC) patients will have brain metastases (BM) at presentation (2), and 25–30% will develop BM during the course of their disease (3). Life-expectancy for these patients is poor, with a median survival of only 3.4 months (4). Moreover, many will suffer considerable loss of autonomy due to neurocognitive and functional deficits, as well as morbidity associated with medications such as steroids and anti-epileptic drugs.

Despite these grim realities, there is room for optimism among identifiable subsets of these patients. A recent published series of NSCLC patients with synchronous BM receiving surgery or radiosurgery to the brain and aggressive management of their extracranial disease reported a median overall survival (OS) of 12.1 months (5). Improved surgical techniques and radiation therapy (RT) technology, as well as more effective systemic treatments and multimodality approaches have led to these superior outcomes. Moreover, renewed hope has emerged from the use of small-molecule drugs targeting oncogenic mutations, which have shown promising activity both extra-cranially and intra-cranially (6).

## Prognostic Factors

Several variables have been established of prognostic importance in determining potential outcomes for patients harboring BM. In 1997, the Radiation Therapy Oncology Group (RTOG) performed a recursive partitioning analysis (RPA) from a historical database of 1200 patients treated with whole-brain radiation therapy (WBRT) from three RTOG BM trials and published a prognostic scoring system (7). Three scoring classes were identified based on patients’ Karnofsky performance score (KPS), age, status of primary tumor, and extent of extracranial disease (Table 1). Median survival ranged from 2.3 months for patients in class III to 7.1 months for those in class I.

**Table 1:** **Prognostic indexes for metastatic brain disease**.

**RECURSIVE PARTITIONING ANALYSIS**	
Class 1	Age < 65; KPS ≥ 70, primary controlled; no extra-cranial disease
Class 2	Patients not in class 1 or 2
Class 3	KPS < 70
**BASIC SCORE FOR BRAIN METASTASES**	
Score 0	KPS 50–70; primary uncontrolled; extra-cranial disease present
Score 1	KPS 80–100; primary controlled; no extra-cranial disease
**SCORE INDEX FOR RADIOSURGERY**	
Score 0	KPS ≤ 50; age ≥ 60; extra-cranial disease progressive; lesions ≥ 3; volume > 13 ml (largest lesion)
Score 1	KPS 60–70; age 51–50; extra-cranial disease stable; lesion 2; volume 5–13 ml
Score 2	KPS > 80; age ≤ 50; systemic disease NED; lesion 1; volume < 5
**GRADED PROGNOSTIC ASSESSMENT**	
Score 0	KPS < 70; age > 60; lesions > 3; extra-cranial disease present
Score 0.5	KPS 70–80; age 50–59; lesions 2–3
Score 1	KPS 90–100; age < 50; lesion 1; no extra-cranial disease

*KPS, Karnofsky performance status; NED, no evidence of disease*.

Since then, several other scoring classifications have been described (4, 8–11) as shown in Table 1. All these classifications have limitations, but are able to consistently prognosticate outcomes based on the defined scoring. Irrespective of the scoring classification used, age, performance status, number of brain lesions, and the presence of extracranial metastases are the variables that better define prognosis. Given the high heterogeneity of the BM patient population, one should not rely exclusively on these indices when assessing the management for such patients. A comparative review of five of these prognostic indexes using an artificial neural network in patients with BM and receiving WBRT (12) suggests that the graded prognostic assessment index (10) was the most powerful in predicting survival.

Increasingly, molecular biomarkers are also being identified with prognostic significance in NSCLC, some with positive [e.g., EGFR (del-19 and L858R)] and others with negative (e.g., ERCC1, BRCA1, TP53, and KRAS) prognostic value (13). In addition, microarray-derived gene signatures provide the potential for even greater prognostic ability (14). However, many of these biomarkers require further validation, and are not yet ready for entry into routine clinical practice.

## Treatment

### Supportive

Early integration of palliative care in the management of metastatic NSLCC has been demonstrated to improve both quality of life and mood, and is associated with improved survival despite less aggressive end of life treatment (15). In addition to general palliative measures, patients with BM often necessitate additional supportive medications such as steroids and anti-seizure medications. Corticosteroids can be vital drugs in the control of intracranial edema from BM and the relief of related symptoms. However, in light of their considerable short- and long-term side effects, steroids should be used judiciously. Hence, a systematic review on the subject (16) has made the following recommendations:

- If corticosteroids are given, dexamethasone is the best choice (level 3).
- Starting doses of 4–8 mg of dexamethasone should be given for temporary relief of symptoms related to increased intracranial pressure. In more severe cases, where symptoms suggest impending herniation, doses of 16 mg/day or more may be considered (level 3).
- There is insufficient evidence to guide treatment recommendations for asymptomatic BM.

### Surgery

Up to few decades ago, surgical resection was mainly used to establish a diagnosis or to alleviate mass-effect symptoms. More recently, its definitive role in improving disease control for patients with single, resectable metastasis has been shown to be significant. Three randomized studies (17–19) have addressed the potential therapeutic value of surgical resection by comparing surgery followed by WBRT vs. WBRT alone in patients with a single brain metastasis (Table 2).

**Table 2:** **Randomized trials of WBRT in brain metastases**.

Author	No. patients	Randomization	Local control	Survival (months)	*p* Value
**WBRT ± SURGERY**					
Patchell (7)	48	WBRT	48%	3.6	*p* < 0.001
		S + WBRT	80%	9.5	
Vecht (8)	63	WBRT	NR	6.0	*p* = 0.04
		S + WBRT		10.0	
Mintz (9)	84	WBRT	NR	6.3	*p* = 0.39
		S + WBRT		5.6	

**Author**	**No. patients**	**Randomization**	**Local control (%)**	**Survival (months)**	**Neurologic death**

**WBRT ± STEREOTACTIC RADIOSURGERY**					
Chougule (23)	73	SRS	87	5	NR
		SRS + WBRT	91	9	
Aoyama (34)	132	SRS	72.5	7.5	19.3%
		SRS + WBRT	88.7	8	22.8%
Chang (35)	58	SRS	67	15.2	NR
		SRS + WBRT	100	5.7	
Kocher (36)	199	SRS	69	10.7	44%
		SRS + WBRT	81	10.9	28%

*No, number; S, surgery; SRS, stereotactic radiosurgery; WBRT, whole brain radiation therapy; NR, not reported*.

In two of these trials (17, 18), a survival benefit was reported for patients undergoing the combined approach. Patchell et al. (17) randomized 48 good-performing (KPS ≥70) patients with an MRI-diagnosed, tissue-proven single lesion to surgical resection plus WBRT (36 Gy in 12 fractions) vs. WBRT alone. Of interest, 11% of patients were excluded because no metastatic disease was seen on the biopsy specimens. The authors reported a statistically significant improvement in survival (median survival: 40 vs. 15 weeks, *p* < 0.01) favoring the combined therapy, as well as a reduction in brain recurrence rates and neurologic death. Vecht at al. (18) compared WBRT (40 Gy in 20 fractions) with the same WBRT preceded by surgery. Similarly, the combined approach showed a survival advantage (median survival: 10 vs. 6 months, *p* = 0.04). In this study, patients were stratified for progressive vs. stable extracranial disease, which proved to be the most important prognosticator for survival.

In contrast, the study by Mintz et al. (19) failed to show a survival benefit when WBRT (30 Gy in 10 fractions) followed surgical resection. The median survival for the WBRT group was 6.3 vs. 5.6 months for the combined modality group (*p* = 0.24). The median survival in the Mintz et al. (19) series was lower than the two other randomized studies and may be explained by the selection of patients with lower KPS or with more extensive extra cranial systemic disease (45% of patients). In addition, MRI was not routinely used to exclude multiple metastases.

It should be mentioned that all of these randomized studies had small patient numbers and did not include relatively radiosensitive tumors such as small cell lung cancer, lymphoma, myeloma, and germ cell tumors. Also, these trials were not specific for NSCLC patients, although this histology was the predominant one in all trials.

Despite these limitations, the current level 1 evidence supports the use of WBRT post-surgical resection in patients with a single, resectable lesion, good performance, and limited extracranial disease. For patients with multiple metastatic lesions, poor performance scores, and extensive systemic disease an evidence-based recommendation for the combined approach cannot be made.

A follow-up trial by Patchell and colleagues (24) addressed the real need of WBRT post-resection of a single brain metastasis. In a multi-center study, 95 patients (60% with NSCLC) with KPS ≥70 undergoing a complete resection of a single brain metastasis were randomized to WBRT (50.4 Gy in 28 fractions) or no further treatment for a primary end-point of tumor recurrence anywhere in the brain. A total of 95 patients were randomized and again NSCLC was the predominant tumor type. The group receiving post-operative WBRT experienced a significantly lower rate of brain recurrence (18 vs. 70%, *p* < 0.001). WBRT also decreased brain recurrence at the site of the original metastasis (10 vs. 41%, *p* < 0.001) and at other sites in the brain (14 vs. 37%, *p* < 0.01). Although OS was not different between groups, importantly, post-operative WBRT significantly prevented death from neurologic causes (14 vs. 44%, *p* = 0.003). This trial defined the need for adjuvant RT post-resection of a single brain metastasis.

## Radiation Therapy

### Whole-brain radiation therapy

The use of WBRT for patients harboring BM is considered by many as the standard treatment. The rationale for treating the whole brain is based on the presumption that micro-metastatic deposits of tumor cells are present elsewhere in the brain. WBRT is the most frequently used treatment for the management of BM and its use is associated with improvement in neurologic symptoms and decreased neurologic death (25). The RTOG and other investigators (26–31) conducted several randomized trials evaluating different dose/fractionation regimens, but no particular regimen appears to be superior in terms of disease control or survival. Typically, a dose of 20 Gy in 5 fractions or 30 Gy in 10 fractions is recommended. Approximately 60% of patients will experience a complete or partial response with a similar rate for symptoms improvement, though usually transient.

One major concern with the use of WBRT is the risk of neurocognitive deficits, particularly short-term memory. Unfortunately, the real rate and magnitude of neurocognitive deficits post-WBRT has not been properly studied. It has been shown that over 90% of patients with BM had impairment in one or more neurocognitive tests at baseline and prior to WBRT (32). Proponents of WBRT argue that it is the disease progression in the brain *not* treated by WBRT that, in fact, compromises the patient’s neurocognitive function. However, some patients develop cognitive problems that cannot be simply explained by disease progression elsewhere in the brain. Late effects from WBRT are usually seen after 6 months post-treatment and are secondary to white matter damage. Considering that many patients will not survive beyond 6 months, it is plausible to consider that cognitive deficits would be seen in larger proportion of patients should they survive longer. For a comprehensive review of the subject, we recommend the paper by McDuff et al. (33).

Recent approaches to reduce the potentially negative effects of WBRT on cognitive function include the concomitant use of memantine (20) and hippocampal sparing during WBRT (21). Memantine, a potential neuroprotector, was used during EBRT in a recent RTOG randomized trial (20). Patients receiving the drug had improved cognitive function in several domains. Gondi et al. (21) presented a phase II RTOG study of hippocampal sparing in patients undergoing WBRT for BM. Although this was a single arm trial, the declines in cognitive function are less than what was observed from historical controls.

### Stereotactic radiosurgery

Stereotactic radiosurgery (SRS) delivers a single high dose of irradiation to the target volume while avoiding the surrounding normal tissues. A randomized trial conducted by the RTOG (22) showed that the addition of SRS to WBRT was superior to WBRT alone in patients with a newly diagnosed single brain lesion. A survival benefit was not seen for patients with two or three metastatic lesions, although local brain control was significantly improved with the addition of SRS. Given its focal delivery of irradiation, there have been concerns that its isolated use could lead to an increased rate of failure elsewhere in the brain. However, concerns with cognitive deficits from WBRT led investigators to use SRS alone in selected patients, reserving WBRT for a later date if necessary.

To address to this question, four randomized trials have, to date, compared SRS alone vs. SRS plus WBRT in patients with a limited number of metastatic lesions (23, 34–36). One of them has only been reported in abstract form (23). Table 2 summarizes the results of these trials.

Despite differences in patient selection and treatment design, all trials consistently show no significant difference in survival, but have shown a significant reduction in intracranial failures and death from brain causes. One study (35) had a neurocognitive end-point – Hopkins Verbal Learning Test (HVLT) – at 4 months post-treatment. This small study was stopped prematurely because an interim analysis showed neurocognitive function at 4 months significantly worse after SRS + WBRT than after SRS alone, although brain control at 1 year was significantly better for the WBRT + SRS arm (73 vs. 27%, *p* = 0.0003). On the other hand, in the Japanese trial (34), there was a significant decline in mini-mental score when SRS was given alone making the authors conclude that BM control was the most important factor for preserving neurocognitive function.

Whether SRS can replace WBRT in newly diagnosed BM remains to be determined and treatment decisions should be individualized taking into consideration the patients’ wishes, age, intra and extracranial disease extent, and prognosis.

### Chemotherapy

Due to the failure of most drugs to cross the intact blood–brain barrier (BBB), the role of chemotherapy in the treatment of BM has been viewed critically (2). Chemotherapy drugs are generally large (>150 kDa), ionized, hydrophilic, and often protein-bound, and therefore, ill-suited to penetrate the tight-junctions, electrochemical barrier, astrocyte foot-processes, and highly regulated transmembrane transport proteins of the central nervous system’s endothelial vasculature (37).

However, the effects of the BBB may be over-estimated. First, there is evidence that the BBB of BM is disrupted, as evidenced by the presence of peritumoral edema and the accumulation of contrast media during computed tomography or magnetic resonance assessments (38, 39). Second, there is evidence of intracranial tumor response, even to drugs that in healthy systems have little central nervous system penetration. In a recent review (37), the response rates (RRs) of BM to platinum-based regimens in seven clinical trials of treatment-naïve NSCLC patients were similar to those achieved extra-cranially, ranging from 30 to 50%. However, the median survival remained only 5–8 months in most cases. In the same review, three trials using temozolomide achieved a RR of only 0–10%, suggesting that the selection of chemotherapy drugs should be based mainly on their established anti-tumor activity to extracranial sites, and not on considerations of BBB penetrance.

More recently, two phase II trials have examined the use of cisplatin and pemetrexed for the treatment of NSCLC with BM. In one trial, 43 chemo-naive NSCLC patients (93% non-squamous histology) with BM received up to six cycles of cisplatin and pemetrexed at standard doses (40). WBRT was given in cases of disease progression or at chemotherapy completion. Cerebral, extra-cerebral, and objective RRs by intention to treat (ITT) were 41.9, 34.9, and 34.9%, respectively. Median OS and progression-free survival (PFS) were 7.4 and 4.0 months, respectively.

In another phase II trial (41), newly diagnosed NSCLC patients with BM received up to six cycles of cisplatin and pemetrexed concurrently with WBRT (30 Gy/10 fractions) during days 1–12 of the first cycle. Among the 41 patients evaluable for response (100% adenocarcinoma), the cerebral, extra-cerebral, and overall RRs were 68.3, 34.1, and 36.6%, respectively. The median PFS of BM and OS were 10.6 and 12.6 months, respectively. The hematologic toxicities were generally mild or moderate and there were no grade 4 or higher non-hematologic toxicities. The combined treatment was generally safe and well-tolerated.

### Targeted therapy

The use of drugs targeting the proteins of mutated EGFR and anaplastic lymphoma kinase (ALK) genes has become standard of care in the systemic treatment of metastatic NSCLC (42). In first-line clinical trials of the EGFR-targeted drugs gefitinib, erlotinib, and afatinib, objective response rates (ORRs) of 55–83% were observed, mostly clustering above 70% (43). In addition, large international phase III trials comparing EGFR tyrosine kinase inhibitors (TKIs) against platinum doublet chemotherapy have achieved significant PFS benefits of >4 months with hazard-ratios (HRs) ranging from 0.37 to 0.58, and improvements to symptoms and quality of life (44–47).

The ALK-inhibitor crizotinib has also demonstrated strong anti-tumor activity systemically. In a phase III second-line NSCLC trial of patients with ALK-rearranged tumors randomized to receive crizotinib vs. chemotherapy with docetaxel or pemetrexed, an ORR = 65% was demonstrated, as well as a PFS benefit of 4.7 months vs. chemotherapy (7.7 vs. 3.0 months, HR 0.49, *p* < 0.001) (48). Similarly, in a phase I study of the newer ALK-inhibitor ceritinib, an ORR = 58% was achieved, including an ORR = 56% in tumors that had progressed on crizotinib (49).

The mutation status of tumors is usually derived from biopsies obtained at extracranial sites, and thus, does not necessarily guarantee a mutation in the sub-clones within the brain. However, a Chinese study of 136 NSCLC patients with resected BM, in which an EGFR mutation was identified in 57% of the BM, found a concordance rate of 93.3% in the EGFR mutation status between the primary tumor and BM (50). This suggests that primary tumor EGFR status is a very good surrogate for EGFR mutation status of the BM. In this same cohort of patients, the median OS was 24.5 months in the EGFR mutation group, compared to 15 months in the wild-type group. This finding is consistent with other studies identifying EGFR mutation status as a positive prognostic factor among patients with BM (51).

Just as targeted therapy with EGFR and ALK inhibitors is highly active systemically among molecularly selected NSCLC patients, there is mounting evidence that this is also true for activity intra-cranially. A recent review has examined the use of the EGFR inhibitors gefitinib and erlotinib in BM among NSCLC patients (6). In the eight phase II clinical trials included in the review, the intracranial RRs with gefitinib were 27–32% in unselected patients, 43% in an Asian population without molecular selection, and 70–89% in molecularly selected patients. Similarly, intracranial RRs were 56 and 82% for erlotinib in clinically and molecularly selected patients, respectively. Taken together, these results highlight both robust intracranial activity and the importance of EGFR mutation status as a predictor of intracranial response. In addition, for the three studies where OS data were presented, the median OS results were 12.9, 18.8, and 19.8 months, respectively.

## Conclusion

The management of patients with BM has evolved over the years from an under-studied area to a field of exciting active research. Supportive therapy, surgery, and RT remain the mainstays of management for these patients. Additional areas of active research include techniques to preserve neurocognitive functions with radiotherapy (20, 52), improving the detection and clinical utility of circulating tumor cells (53), and novel systemic approaches including immunotherapy alone (54, 55) or in combination with radiotherapy (56), anti-metabolic agents (57), anti-angiogenesis drugs (58), and novel targeted therapies for a growing list of oncogenic mutations (59). Ultimately, the optimal management strategy will employ a multi-disciplinary approach accounting for individual characteristics of both patient and tumor.

## Conflict of Interest Statement

The authors declare that the research was conducted in the absence of any commercial or financial relationships that could be construed as a potential conflict of interest.
